# Bacterial diversity significantly reduces toward the late stages among filarial lymphedema patients in the Ahanta West District of Ghana: A cross‐sectional study

**DOI:** 10.1002/hsr2.724

**Published:** 2022-07-20

**Authors:** Samuel O. Asiedu, Priscilla Kini, Bill C. Aglomasa, Emmanuel K. A. Amewu, Ebenezer Asiedu, Solomon Wireko, Kennedy G. Boahen, Afiat Berbudi, Augustina A. Sylverken, Alexander Kwarteng

**Affiliations:** ^1^ Department of Theoretical and Applied Biology Kwame Nkrumah University of Science and Technology Kumasi Ghana; ^2^ Kumasi Centre for Collaborative Research in Tropical Medicine Kwame Nkrumah University of Science and Technology Kumasi Ghana; ^3^ Department of Laboratory Technology Kumasi Technical University Kumasi Ghana; ^4^ Department of Microbiology School of Medical Sciences Kwame Nkrumah University of Science and Technology Kumasi Ghana; ^5^ Division of Parasitology, Department of Biomedical Sciences, Faculty of Medicine Universitas Padjadjaran Bandung Indonesia; ^6^ Department of Biochemistry and Biotechnology Kwame Nkrumah University of Science and Technology Kumasi Ghana

**Keywords:** antimicrobial resistance, lymphatic filariasis, lymphedema, MALDI‐TOF, microbiome

## Abstract

**Background:**

Lymphatic Filariasis (LF), a neglected tropical disease, has been speculated to be complicated by secondary bacteria, yet a systematic documentation of these bacterial populations is lacking. Thus, the primary focus of this study was to profile bacteria diversity in the progression of filarial lymphedema among LF individuals with or without wounds.

**Methods:**

A cross‐sectional study design recruited 132 LF individuals presenting with lymphedema with or without wounds from eight communities in the Ahanta West District in the Western Region, Ghana. Swabs from the lymphedematous limbs, ulcers, pus, and cutaneous surfaces were cultured using standard culture‐based techniques. The culture isolates were subsequently profiled using Matrix‐assisted Laser Desorption/Ionization Time of Flight Mass Spectrometry.

**Results:**

Of the 132 LF participants recruited, 65% (85) had filarial lymphedema with no wounds. In total, 84% (235) of the bacterial isolates were identified. The remaining 16% (46) could not be identified with the method employed. Additionally, 129(55%) of the strains belonged to the phylum Firmicutes, while 61 (26%) and 45 (19%) represented Proteobacteria and Actinobacteria, respectively. Generally, irrespective of the samples type (i.e., wound sample and non‐wound samples), there was a sharp increase of bacteria diversity from Stages 1 to 3 and a drastic decrease in these numbers by Stage 4, followed by another surge and a gradual decline in the advanced stages of the disease. The Shannon Diversity Index and Equitability for participants with and without wounds were (3.482, 0.94) and (3.023, 0.75), respectively. Further, *Staphylococcus haemolyticus* and *Escherichia coli* showed resistance to tetracycline, chloramphenicol, and penicillin.

**Conclusion:**

The present study reveals a sharp decline in bacterial load at the late stages of filarial lymphedema patients. In addition, we report an emerging antimicrobial resistance trend of *S. haemolyticus* and *E. coli* against commonly used antibiotics such as tetracycline, chloramphenicol, and penicillin in communities endemic for LF in the Ahanta West District, Ghana. This could pose a huge challenge to the management of the disease; particularly as current treatments are not quite effective against the infection.

## 1 BACKGROUND

The human skin is regarded as the largest organ and mainly serves as a protective barrier against biological, mechanical, and physical agents and is home for many microbes.^1^ Recent studies have suggested that these microbes (archaea, bacteria, fungi, and viruses) play crucial roles in maintaining a healthy balance of human physiology, and thus, any significant change in their composition may lead to a disease state.^2^ For instance, scientific evidence suggests that a substantial change in the microbiota could possibly be the reason for many skin infections such as atopic dermatitis, psoriasis, hidradenitis suppurativa, and acne.^3^ This knowledge has garnered the idea of manipulating microbial communities in treating illness.^4^ Thus, the NIH Common Fund Human Microbiome Project (HMP) established in 2008 to characterize the human microbiome and analyze its role in human health and disease, has made substantial strides in decoding the exact functional roles of the human microbiome, particularly the skin microbiome in diseases.^5^, ^6^

Despite increasing evidence of the role of microbiota in diseases, there is a dearth of information on the role of the microbiome in neglected tropical diseases, especially diseases which affect the skin. Lymphatic filariasis (LF) has been hypothesized to be complicated by secondary bacteria, which aggravate the occasional severe pain people living with the infection experience.^7^, ^8^, ^9^ Moreover, bacteriological study on the skin biopsy, lymph node, lymph showed populations of *Staphylococcus epidermis*, *S. hominis*, Micrococcus species and *luteus*, *Corynebacterium*, *Streptococcus acidominus* which hitherto are sterile sites.^8^ More specifically, there has been a report of direct link between the group A streptococci infection with acute dermatolymphangioadenitis (ADLA) as a higher tire of anti‐streptolysin O (ASO) was observed to positively correlate with ADLA experience by LF patients.^10^ This observation has been suggested to be due to the Gram‐positive bacteria (group A streptococci) invading the skin barrier into the peripheral blood.^11^, ^12^

LF is documented to infect nearly 120 million individuals worldwide with almost 40 million individuals currently suffering from varying degrees of disability and morbidity.^13^ Presently, Filariasis Test Strip (FTS) ^14^ is used to diagnose LF after several years of using the thick smear technique to detect the presence of microfilariae.^15^

Lymphedema is the main sequelae of the infection affecting about 2% of the population of LF endemic areas.^16^ Lymphedema is estimated to affect 15 million persons living with the disease.^13^ However, studies have attributed the progression of the lymphedema of the leg to recurrent episodes of ADLA and the presence of lesions with varying sizes.^9^, ^17^

Lesions or wounds of LF individuals have been observed to be located mainly on the interdigital part of the feet with few dotted around the lower extremities.^9^ These wounds serve as the entry points of secondary bacterial (microbial infection); thus, complicating the morbidity of LF patients.^17^ Entry lesions in filarial lymphedema individuals are wounds located on the skin of affected limb, which function as entries for pathogens,^18^ but these lesions can also be located on limbs without lymphedema. These wounds are mostly chronic, which tend to promote the growth of fungi, thus exacerbating the ADLA episodes leading to excruciating pain in patients. Considering that the second objective of the Global Programme for Elimination of Filariasis (GPELF) is to alleviate pain and morbidity among LF patients. Despite speculating possible secondary bacteria complicating LF, systematic documentation of these bacterial populations is limited.

Additionally, there is a growing concern of antimicrobial resistance of microbes, particularly as there is limited data on surveillance of antimicrobial resistance in sub‐Saharan Africa.^19^, ^20^ From our field observation of LF endemic communities in the Ahanta West District of Ghana, affected individuals routinely use antibiotics when they have “filarial attacks,” and some use it topically on their wounds. Thus, profiling the antimicrobial resistance and susceptibilities of isolated bacteria from these LF individuals is crucial.

In this study, we used conventional culture methods augmented with Matrix‐assisted Laser Desorption/Ionization Time of Flight (MALDI‐TOF) technology to understand bacteria diversity in the progression of filarial lymphedema among LF individuals with or without wounds.

## 2 MATERIALS AND METHODS

### 2.1 Study design

**Figure 1:**
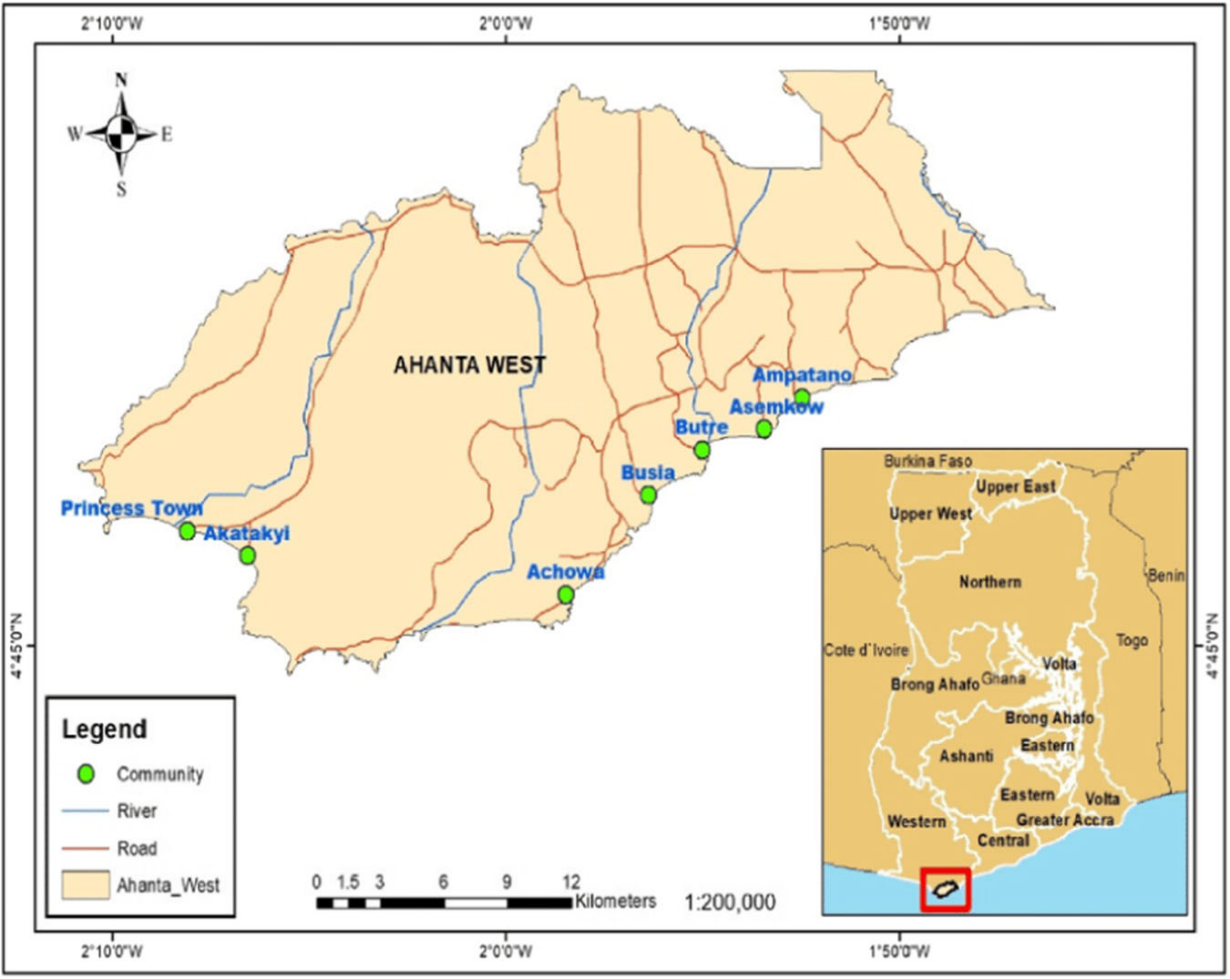
A geographical map of the study communities

This study was cross‐sectional carried out in eight (8) LF‐endemic communities in the Ahanta West District, Ghana. Identification of microbial populations was performed as previously described.^21^ The study communities were Akatakyi, Princess Town, Dixcove, Ampatano, Asemkow, Butre, Achowa, and Busia, which are LF hotspots located at the Western Region of Ghana (Figure 1).

### 2.2 Study participants

Individuals presenting with filarial lymphedema with or without wounds and had resided in the community for the past ten (10) years were potential participants for the study. Participants were screened by experienced filarial experts and registered for the study if they gave their informed consent after thorough explanation of the study protocols in the local dialects were recruited onto the study. The study was approved by the Committee for Human Research, Publications, and Ethics (CHRPE/AP/649/19) at the School of Medical Sciences, Kwame Nkrumah University of Science and Technology, Kumasi, Ghana.

### 2.3 Data collection

Structured questionnaires were used to obtain socio‐demography information of the study participants. Information about the pathology, such as the stage of the leg (severity of the disease), location of the wound and wound appearance was also obtained. Wound swab or the skin swab of the lymphedematous leg was collected. Here, those presenting with wounds had their wounds and their edges cleaned with sterile saline or water. The Levine method of sample collection was implored as previously described.^22^ All samples collected under aseptic conditions (i.e., clean work surface, perform hand hygiene with soap and water, etc.) and kept in Amine transport medium (Copan Diagnostics Inc.) which were immediately transferred into a liquid nitrogen tank (−120°C) and transported to the Kumasi Centre for Collaborative Research in Tropical Medicine (KCCR) laboratory, Kumasi, for further laboratory analysis.

### 2.4 Bacterial culturing

To determine the bacteria composition of the swab samples, the wound or skin swabs were plated on both Columbia nalidixic acid (CNA) agar supplemented with 5% Sheep and MacConkey agar (Mac). Under aerobic and anaerobic conditions, the samples were cultured on the respective media and then incubated overnight at 35–37°C. The pure culture isolates from CNA and Mac were preserved in cryotubes with brain heart infusion (BHI) medium and stored at −80°C for later identification.

### 2.5 Bacterial identification

To identify the bacterial isolates in the swab samples, the culture isolates were typed using MALDI‐TOF technology (Bruker MALDI Biotyper, Server Version: 4.1.31 (SIRO) 314 2015‐09‐30_17‐53‐06). The isolates saved in BHI media in the cryotubes at −80°C were thawed and placed in an incubator for about 15 min before plating them on the media to ensure maximum growth of the isolates. The identification of bacteria isolates was done using the manufacturer's manual.^23^

### 2.6 Antimicrobial susceptibility testing

To ascertain the susceptibility or resistance of strains to some selected antibiotics, antimicrobial susceptibility testing was done by disk diffusion technique according to guidelines and breakpoints of the Clinical Laboratory Standards Institute.^24^ Here, a sterile loop was used to touch 3–5 of the bacterial strains on the culture medium and emulsified in 3–4 ml of sterile physiological saline. The turbidity of bacterial suspension was then compared with the turbidity standard (0.5 McFarland Standard). The sterile swab was dipped into the bacterial suspension and then plated on Mueller Hinton (MH) agar except when the strain was a fastidious organism, which was rather plated on blood agar (BA) or chocolate agar (CA). The swab stick dipped in the bacterial suspension was streaked evenly over the surface of the medium in three directions, rotating the plate approximately 60° to ensure even distribution. With the petri dish lid in place, 3–5 min was allowed for the surface of the agar to dry. A multidisc dispenser was used to place the appropriate antimicrobial discs (ampicillin 10 µg, erythromycin 15 µg, tetracycline 30 µg, gentamycin 10 µg, ciprofloxacin 30 µg, clindamycin 30 µg, cefoxitin 30 µg, chloramphenicol 30 µg, penicillin 10 µg, and trimethoprim/sulfamethoxazole (1.25/23.75 µg) from Oxoid. Each disc was lightly pressed down to ensure its contact with the agar. The plates were then inverted and incubated aerobically at 35°C for 16–18 h. Using a meter ruler on the underside of the plate, the diameter of each zone of inhibition of the antimicrobial disc was measured in millimeters (mm). Each set of tests was controlled using susceptible *Staphylococcus aureus* (ATCC 25923) for Gram‐positive bacteria and *Escherichia coli* (ATCC 25922) for Gram‐negative bacteria.

### 2.7 Statistical analysis

The data obtained from the structured questionnaires were entered into Epi Info Version 7.2.3.0 and exported into a Microsoft Excel sheet for further analysis. Descriptive analysis was performed to generate the proportion of bacterial populations. Python 3.7 was used to analyze the distribution and the frequency of the drug‐resistant or sensitive strains and the clustering of bacterial strains based on the study communities. A one‐way analysis of variance (ANOVA) was used to determine any statistical difference between the distribution of bacterial strains among the study communities. Any result with a *p *value less than 0.05 was considered statistically significant.

## 3 RESULTS

### 3.1 Demography of study participants

**Figure 2:**
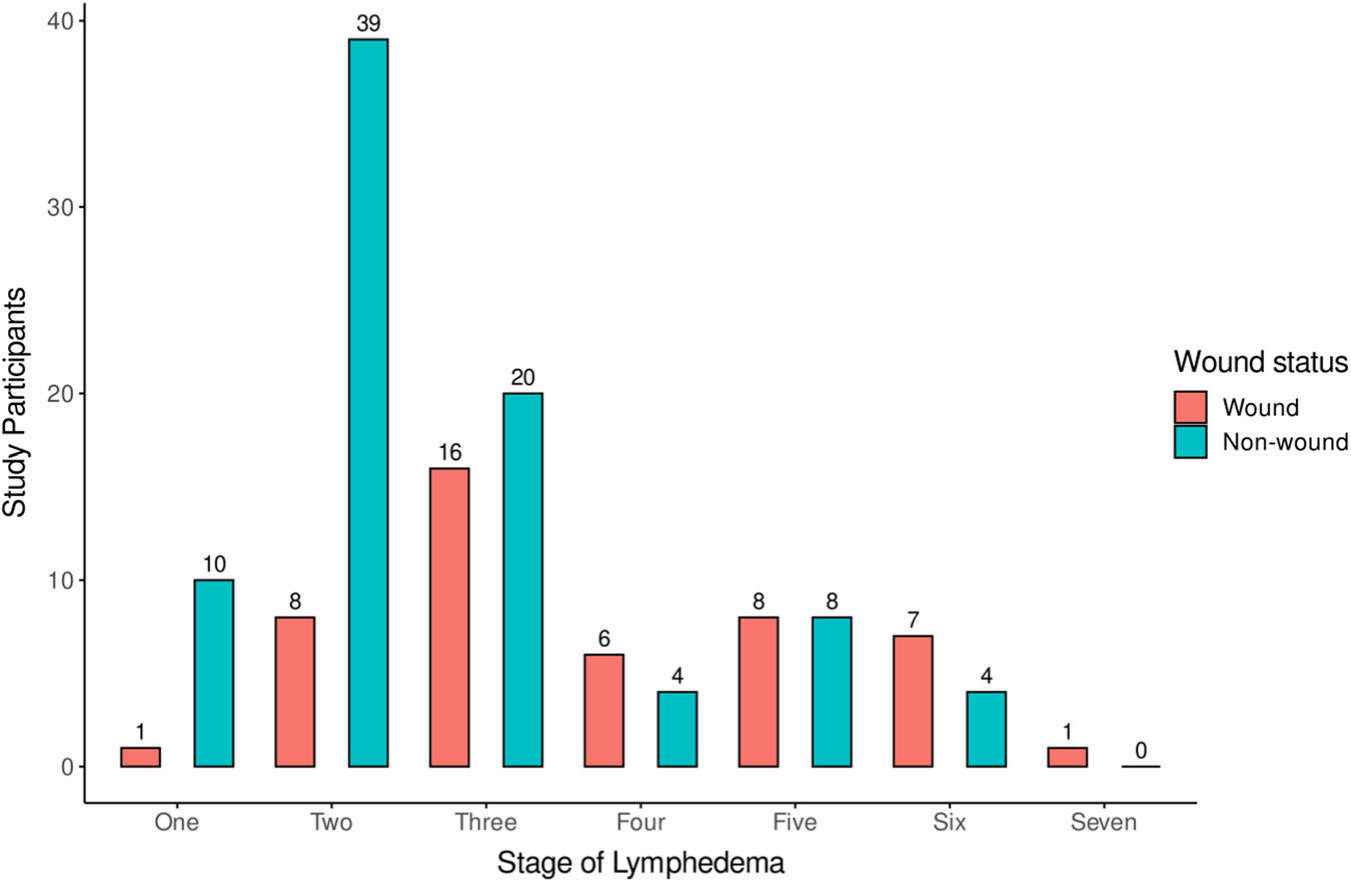
The leg staging of lymphedema presented by study participants (Leg staging according to ref. [25]

In total, 132 LF participants were recruited for the study from eight (8) communities in the Ahanta West District. Of these, 93 (70%) were females. The age range of the participants was 18–81 years, with a median of 48 years (Table 1). In addition, a greater proportion (69/85) of the study participants without wounds on their lymphedematous legs presented with lower stages of lymphedema (i.e., Stages 1–3), with Stage 2 being the highest. Among 47 individuals with wounds on their legs, 22 were presented with the advanced stages of lymphedema (Figure 2).

**Table 1:** Demography of study participants

Characteristics	No. (%)
**Age**	
<21	6 (4.5)
21–30	7 (5.3)
31–40	26 (19.7)
41–50	58 (43.9)
51–60	25 (18.9)
>60	10 (7.6)
**Median age**	48
**Age range**	18–86
**Gender**	
Male	39 (29.5)
Female	93 (70.5)
**Community**	
Princess Town	18 (13.6)
Akatakyi	26 (19.7)
Ampatano	20 (15.2)
Asemkow	20 (15.2)
Busua	13 (9.8)
Butre	19 (14.4)
Dixcove	12 (9.1)
Achowa	4 (3.0)
**Occupation**	
Agrarian activities	66 (50.0)
Petty trading	46 (34.8)
Unemployed	20 (15.2)

### 3.2 Bacterial strains identified among the study participants

**Figure 3:**
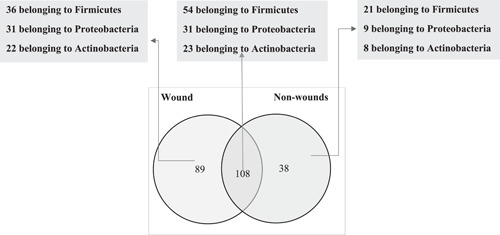
A Venn diagram of bacterial communities in the wound and non‐wound samples

In all, 281 pure bacterial isolates were cultured, of which 235 (84%) were identified using the MALDI‐TOF identification platform. Of the 235 bacteria identified, the most occurring bacterial genus was *Staphylococcus*, representing 59(25%) of all the strains. The bacterial composition of the study participants with wounds and those without wounds samples differed. Out of the 235 bacterial isolates identified, 108 were observed in both wounds and non‐wound samples, while 89 and 38 strains were exclusively identified in wounds and non‐wound samples, respectively (Figure 3). Moreover, there were 59 distinct isolates with 11 isolates from non‐wound, 27 isolates from wounds and 21 isolates were common in both sample types. There were more prominent potential pathogens such as *Pseudomonas aeruginosa*, *Klebsiella pneumoniae* isolated from individuals presenting with the wound than those without wounds. Likewise, there were higher proportions of Gram‐negative bacteria in the wound sample type than in non‐wounds sample type (Table 2).

**Table 2:** Bacteria isolates classified by sample types

Wounds	Wound and non‐wound	Non‐wounds
*Acinetobacter haemolyticus*	*Achromobacter xylosoxidans*	*Enterobacter aerogenes*
*Acinetobacter junii*	*Acinetobacter baumannii*	*Kocuria marina*
*Aeromonas caviae*	*Bacillus pumilus*	*Leuconostoc lactis*
*Alcaligenes faecalis*	*Burkholderia cenocepacia*	*Providencia rettgeri*
*Arcanobacterium haemolyticum*	*Citrobacter freundii*	*Rhodococcus pyridinivorans*
*Arthrobacter cumminsii*	*Corynebacterium amycolatum*	*Staphylococcus capitis*
*Bacillus cereus*	*Corynebacterium sp*	*Staphylococcus pasteuri*
*Brevundimonas diminuta*	*Corynebacterium striatum*	*Staphylococcus saprophyticus*
*Corynebacterium diphtheriae*	*Enterobacter cloacae*	*Staphylococcus sciuri*
*Cronobacter sakazakii*	*Enterococcus faecalis*	*Staphylococcus simulans*
*Enterobacter asburiae*	*Escherichia coli*	*Staphylococcus xylosus*
*Enterococcus casseliflavus*	*Klebsiella pneumoniae*	
*Globicatella sanguinis*	*Micrococcus luteus*	
*Kerstersia gyiorum*	*Micrococcus lylae*	
*Lactobacillus plantarum*	*Proteus mirabilis*	
*Morganella morganii*	*Staphylococcus epidermidis*	
*Proteus penneri*	*Staphylococcus haemolyticus*	
*Providencia stuartii*	*Staphylococcus hominis*	
*Pseudomonas aeruginosa*	*Staphylococcus warneri*	
*Pseudomonas stutzeri*	*Streptococcus pyogenes*	
*Staphylococcus aureus*		
*Staphylococcus caprae*		
*Staphylococcus lugdunensis*		
*Stenotrophomonas maltophilia*		
*Streptococcus agalactiae*		
*Streptococcus dysgalactiae*		
*Wohlfahrtiimonas chitiniclastica*		

**Figure 4:**
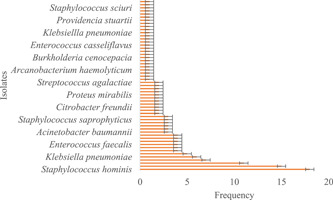
The profile of bacteria isolates among the female study participants

**Figure 5:**
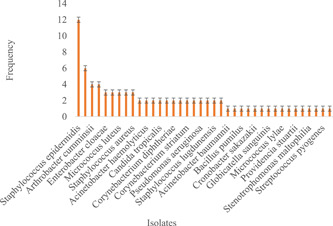
The profile of bacteria isolates among the male study participants

Correspondingly, there was higher bacterial diversity in the wound samples than in the non‐wound samples (Shannon Diversity Index and Equitability) observed in the wound samples was 3.482, 0.94, and 3.023, 0.75 in non‐wound samples. Additionally, when the isolates were classified on gender, there was a higher bacterial diversity among the female participants (Shannon Diversity Index = 3.561, Equitability = 0.89) as compared to the male study participants (Shannon Diversity Index = 3.381, Equitability = 0.90). In all, there were 152 bacteria isolates associated with the female study participants with *Staphylococcus hominis* being the prominent (Figure 4) while 83 bacteria were isolated from the male study participants with *S. epidermis* being the common isolates (Figure 5). Moreover, 129 (55%) of all the bacterial strains identified belonged to the phylum Firmicutes, with the remaining proportions shared between the phyla Proteobacteria (61, 26%) and Actinobacteria (45, 19%). A one‐way ANOVA indicated a significant statistical difference between the phyla of bacteria strains (*p* = 0.000; Table 3).

**Table 3:** A one‐way analysis of variance of the bacteria phylum

	Df	Sum_sq	Mean_sq	*F*	*p* Value
C (Phylum)	2.0	608.857143	304.428571	10.468886	0.000
Residual	18.0	523.428571	29.079365		

### 3.3 The bacteria populations among study participants

**Figure 6:**
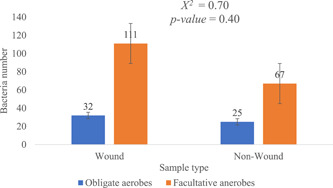
The bacterium types in the non‐wound and wound sample types

To determine the various bacterium types that consist of the microbial population of the study participants, the bacteria typed from the bacteria cultures were further grouped based on how they respond to oxygen. There were generally higher facultative anaerobes (178, 76%) in both wound and non‐wound samples than obligate aerobes (57, 24%). Similarly, 62% (111/178) of the facultative anaerobes were observed in wound samples, with the remaining 38% found in the non‐wound samples. However, there was no significant difference in the type of bacteria among the sample types (*X* ^2^ = 0.70, *p* Value = 0.40; Figure 6).

**Figure 7:**
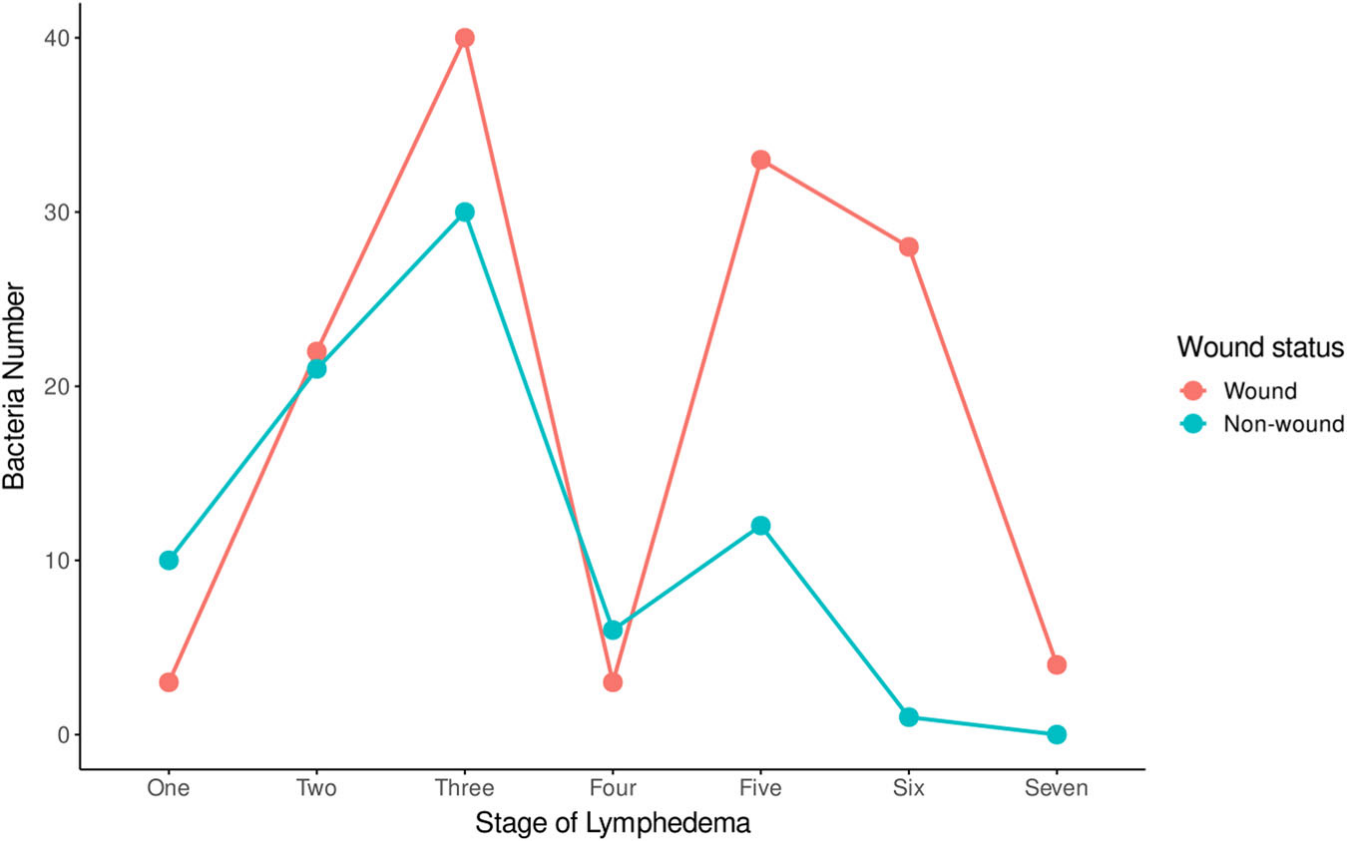
The dynamics of bacteria numbers among study participants

**Figure 8:**
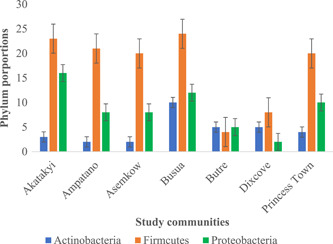
The phylum distribution in the study communities

To ascertain the changes in bacteria numbers during the progression of the disease, a line graph of bacteria numbers and the stage of lymphedema was plotted. Generally, irrespective of the sample type (i.e., wound, and non‐wound samples), there was a sharp increase in bacteria numbers from Stages 1 to 3 and a drastic decrease in these numbers by Stage 4, followed by another surge and a gradual decrease in the advanced stages of the disease (Figure 7). Nevertheless, LF study participants presenting with Stage 3 of lymphedema recorded the highest bacteria numbers irrespective of the sample type (non‐wound sample: 30; wound sample: 40), while the advanced stages of the lymphedema (i.e., Stages 6 and 7) observed lower bacteria numbers (non‐wound sample: 1; wound sample: 4). In addition, the phylum representations of bacteria strains among the various study participant's communities did not differ. As a trend of phylum Firmicutes was observed to be in higher numbers in most study communities followed by Proteobacteria and then Actinobacteria (Figure 8).

### 3.4 Antimicrobial susceptibility testing among bacteria strains in wound sample

**Figure 9:**
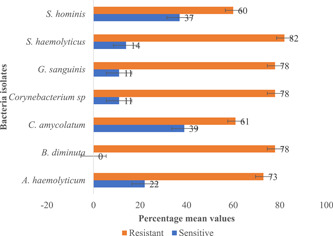
The resistance profile of prominent Gram‐positive bacteria in wound samples

Twenty‐three (23) different bacteria genera were identified as Gram‐positive bacteria from the wound samples. Seven (7) of the Gram‐positive species showed an average of 60% resistance against nine (9) different antibiotics, with *Staphylococcus haemolyticus* showing the highest resistance (82%), followed by *Brevundimonas diminuta*, *Globicatella sanguinis*, and *Corynebacterium* spp., all revealing an average resistance of 78% to the antibiotics (Figure 9). Moreover, the highest frequency of resistance was found against tetracycline (77%), penicillin (76%), and chloramphenicol (71%) among the 78 Gram‐positive bacteria identified from the wound samples (Table 4).

**Table 4:** Antibiotic susceptibility testing

Gram type	Antibiotic	Sample type	*S* (%)	*R* (%)	*I* (%)	*X* ^2^	*p* Value
Gram‐positive bacteria	Chloramphenicol	Wound samples	21 (27)	55 (71)	3 (12)	0.948	0.623
		Non‐wound sample	19 (26)	54 (73)	1 (1)		
	Clindamycin	Wound samples	32 (41)	37 (47)	9 (12)	2.632	0.268
		Non‐wound sample	19 (26)	54 (73)	1 (1)		
	Ciprofloxacin	Wound samples	21 (27)	52 (67)	5 (6)	27.136	0.000
		Non‐wound sample	51 (69)	20 (27)	3 (4)		
	Tetracycline	Wound samples	12 (15)	60 (77)	6 (8)	1.183	0.554
		Non‐wound sample	16 (22)	54 (73)	4 (5)		
	Trimethoprim‐Sulfamethoxazole	Wound samples	21 (27)	50 (64)	7 (9)	3.840	0.147
		Non‐wound sample	31 (42)	37 (50)	6 (8)		
	Erythromycin	Wound samples	32 (41)	37 (47)	9 (12)	29.292	0.000
		Non‐wound sample	39 (53)	27 (36)	8 (11)		
	Gentamicin	Wound samples	48 (62)	22 (28)	8 (10)	24.293	0.000
		Non‐wound sample	48 (65)	20 (27)	6 (8)		
	Penicillin	Wound samples	17 (22)	59 (76)	2 (3)	0.898	0.638
		Non‐wound sample	21 (28)	51 (69)	2 (3)		
	Cefoxitin	Wound samples	33 (42)	43 (55)	2 (3)	7.114	0.029
		Non‐wound sample	47 (64)	25 (34)	2 (3)		
Gram‐negative bacteria	Ampicillin	Wound samples	11 (25)	3 (7)	30 (68)	1.011	0.603
		Non‐wound sample	3 (23)	0 (0)	10 (77)		
	Chloramphenicol	Wound samples	9 (20)	1 (2)	34 (77)	7.035	0.030
		Non‐wound sample	7 (54)	1 (8)	5 (38)		
	Clindamycin	Wound samples	17 (39)	8 (18)	19 (43)	6.520	0.038
		Non‐wound sample	10 (77)	0 (0)	3 (23)		
	Ciprofloxacin	Wound samples	32 (73)	3 (7)	9 (20)	3.227	0.199
		Non‐wound sample	6 (46)	2 (15)	5 (38)		
	Amoxicillin	Wound samples	15 (34)	5 (11)	24 (55)	1.884	0.390
		Non‐wound sample	4 (31)	0 (0)	9 (69)		
	Tetracycline	Wound samples	17 (39)	1 (2)	26 (59)	0.632	0.729
		Non‐wound sample	4 (31)	0 (0)	9 (69)		
	Trimethoprim‐Sulfamethoxazole	Wound samples	11 (25)	7 (16)	26 (59)	2.288	0.319
		Non‐wound sample	6 (46)	1 (8)	6 (46)		
	Gentamicin	Wound samples	30 (68)	3 (7)	11 (25)	1.015	0.602
		Non‐wound sample	9 (69)	0 (0)	4 (31)		
	Ceftriaxone	Wound samples	23 (52)	8 (18)	13 (30)	0.957	0.620
		Non‐wound sample	7 (54)	1 (8)	5 (38)		
	Cefuroxime	Wound samples	14 (32)	3 (7)	27 (61)	6.094	0.047
		Non‐wound sample	9 (69)	0 (0)	4 (31)		
	Ceftazidime	Wound samples	34 (77)	3 (7)	7 (16)	3.583	0.167
		Non‐wound sample	13 (100)	0 (0)	0 (0)		

**Figure 10:**
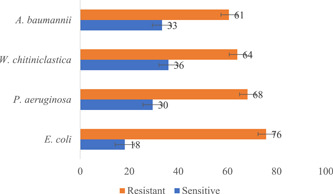
The resistance profile of prominent Gram‐negative bacteria in wound samples

Likewise, 21 different Gram‐negative genera were observed in the wound samples, with 4 showing an average resistance of >60% against 11 antibiotics. *E. coli* showed an average of 76% resistance against the 11 antibiotics, followed by *P. aeruginosa* (68%), *Wohlfahrtiimonas chitiniclastica* (64%), and *Acinetobacter baumannii* (61%; Figure 10). Furthermore, chloramphenicol was observed to be the most resistant to the 44 Gram‐negative bacteria identified in the wound samples (Table 4).

### 3.5 Antimicrobial susceptibility testing among bacteria strains in non‐wound sample

**Figure 11:**
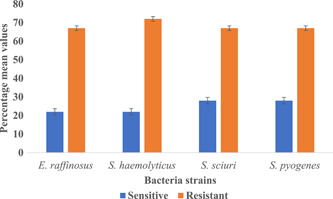
The resistance profile of prominent Gram‐positive bacteria in non‐samples

In all, 74 Gram‐positive bacteria were identified in the non‐wound samples and screened for antimicrobial susceptibility testing against nine different antibiotics. The results showed that four different bacteria species (i.e., *Enterococcus raffinosus*, *S. haemolyticus*, *Staphylococcus sciuri*, and *Streptococcus pyogenes*) showed an average resistance (>60%) against the antibiotics (Figure 11). However, chloramphenicol (73%) and tetracycline (73%) were observed to be the common antibiotics that the Gram‐positive strains were resistant to in the non‐wound samples followed by penicillin (69%). It is also worth noting that Gram‐positive bacteria in the non‐wound samples were more sensitive to ciprofloxacin (69%) than any other antibiotic (Table 4).

**Figure 12:**
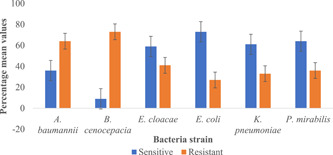
The resistance profile of prominent Gram‐negative bacteria in non‐wound sample

In addition, 13 Gram‐negative bacteria were identified among the non‐wound sample type from 10 different genera. *Burkholderia cenocepacia and A. baumannii* were the only Gram‐negative that showed an average resistance of >60% to the 11 antibiotics (Figure 12). 77% of Gram‐negative bacteria were resistant to ampicillin, followed by amoxicillin (69%) and tetracycline (69%). Generally, most of the Gram‐negative strains (6/13) were sensitive (i.e., sensitivity greater than 50%) to all the antibiotics with all the Gram‐negative bacteria (*n* = 13, 100%) sensitive to ceftazidime. Here, 77% of Gram‐negative strains were sensitive to clindamycin, followed by cefuroxime (69%) and gentamicin (69%; Table 4).

## 4 DISCUSSION

Most of the efforts aimed at eliminating LF have been centered on using the pathogenesis of the filarial nematode and novel or existing therapeutics to aid in interrupting the transmission of diseases, especially in endemic communities.^26^ Nonetheless, speculation of a possible secondary bacteria in complicating the disease have been reported in certain endemic regions, yet systematic documentation of these bacterial population is lacking. This study observed Actinobacteria, Firmicutes, and Proteobacteria as the main bacteria groups present in the wound and non‐wound samples from LF patients in rural Ghana. However, there were greater proportions of the bacteria population belonging to the Firmicutes compared to “normal” skin microbiota of healthy individuals, where Actinobacteria is the most abundant phyla.^27^ This shift in the bacteria population of the skin microbiome of the LF subjects in this study has been reported in other skin diseases such as psoriasis.^28^ Staphylococci species were the most abundant genera (25% of all bacterial isolates in the study) in the phyla Firmicutes. The change of phyla representation of bacteria in this study is not clear, however, the high abundance of staphylococci species indicates the observed changes between the skin of healthy individuals and LF individuals with lymphedema. Staphylococci species, like any other Gram‐positive bacteria, tend to colonize moist surfaces and produce antimicrobial peptides that inhibit the growth of other microorganisms.^29^ For instance, *S. epidermis* has been found to produce phenol‐soluble modulins which inhibit the growth of *S. aureus*.^30^ This assertion could be the reason for higher numbers of *S. epidermis* (*n* = 23) compared to *S. aureus* (*n* = 4) in this study.

This study reveals a clear pattern of changes in the bacteria numbers at the various stages of lymphedema. The general decrease in bacteria numbers toward the late stages of infection could be due to a selective predominance of a few bacteria, which suppress the growth of other bacteria, leading to a reduction in bacterial diversity. Nonetheless, higher bacterial diversity observed in the wounds compared with those without wounds could be due to the moist, warm, and nutritious environment in these chronic wounds, hence a support for polymicrobial colonization. This polymicrobial population in chronic wounds could potentially lead to bacteremia and septicemia,^29^ which was previously reported among LF patients experiencing filarial attacks.^17^ Additionally, polymicrobial colonization has been previously studied to contribute to the chronicity of wounds.^31^

Although a direct correlation between wound chronicity and bacteria isolates was not done in this study, the high prevalence of pathogenic bacteria such as *K. pneumoniae*, *Corynebacterium diphtheriae*, *P. aeruginosa*, and *E. coli* in the wounds of the study participants reflects similar bacteria colonization conducted on diabetic foot ulcers.^32^ However, it is not clear, whether these pathogenic bacteria in these wounds of the study participants originate from the surrounding skin flora or the environment. It has been shown that wound bacteria colonization is frequently part of normal skin microbial flora and through the formation of biofilms, horizontal gene transfer, and mutation, they may become virulent.^33^

This study observed a proportion of anaerobic bacteria among LF patients presenting with wounds. Anaerobes have been documented to play a crucial role in the deterioration of wounds, particularly as it tends to form synergy with aerobes in the wound microbiome.^31^ Anaerobes that colonize the lymphedematous legs can withstand harsh environmental conditions (seawater, frequent rainfalls, etc.), such as where the study participants are located around coastal regions of Ghana. This assertion is further corroborated by the uniform distribution of the phyla representation of the isolated bacteria among the study communities.

There were higher numbers of Gram‐positive bacteria isolated than Gram‐negative bacteria. This finding is not surprising as the human skin microbiota in particularly drier sites as the legs are generally dominated by Gram‐positive bacteria either under the phylum Actinobacteria or Firmicutes.^29^, ^34^ Yet, there were higher numbers of Gram‐negative bacteria in the wound samples (*n* = 44) than those in non‐wound samples (*n* = 13) with *K. pneumoniae* and *P. aeruginosa* as the abundant Gram‐negative bacteria, particularly in the wounds. Reports of Gram‐negative bacteria prevalence in wounds have been documented.^35^, ^36^ However, there is a paucity of information on the predominance of Gram‐positive bacteria in LF wounds. The presence of pathogens such as *K. pneumoniae* and *P. aeruginosa* in wounds has been suggested not to have clinical relevance.^37^

Gram‐positive bacteria are the major cause of nosocomial and community‐acquired infections,^38^ and most antimicrobial agents target this group of bacteria. In this study, we report a trend of resistance of Gram‐positive bacteria against tetracycline, chloramphenicol, and penicillin. Even though methicillin‐resistant *S. aureus* and vancomycin‐resistant enterococci are of great concern for public health institutions such as WHO,^39^ as our group recently reported the increasing reports of resistant Gram‐positive bacteria to broad‐spectrum antibiotics such as penicillin and tetracycline should equally be of concern.^21^

These broad‐spectrum antibiotics are usually the first line for individuals living with LF pathologies in endemic communities in Ghana, as these are the mainstay medications during the painful episodes of filarial attacks. Among the Gram‐positive bacteria isolated in this study; it was observed that *S. haemolyticus* showed the highest resistance to the antimicrobial agents tested. Among coagulase‐negative staphylococci, *S. haemolyticus* has shown a high level of antimicrobial resistance.^40^, ^41^ Next, we observed chloramphenicol, ampicillin, amoxicillin, and tetracycline as the common antibiotics to which Gram‐negative bacteria such as *E. coli* and *B. cenocepacia* were resistant.

The *E. coli* isolated from the lymphedema patients without wounds were sensitive to standard antibiotics used in this study. This observation, however, is not uncommon as some commensal *E. coli* tend to gain virulence factors (via. horizontal transfer of gene) and resistance genes in immunocompromised people such as LF individuals (particularly those with the late stages of the infection and with wounds on their limbs). The pattern of resistance of the Gram‐negative bacteria to these antibiotics has also been reported.^42^ Christopher et al.^43^ observed 90% and 65% of Gram‐negative resistance to tetracycline and chloramphenicol, respectively. Moremi et al.^38^ reported multi‐drug resistant Gram‐negative bacteria from ulcers on the lower limbs with higher resistance against ampicillin (95%, *n* = 171/1810) amoxicillin (83.9%, *n* = 151/180).

These reports could affect the treatment of wounds with antibiotics as a significant proportion of isolates from wound cultures has been observed to show resistance to first‐line antimicrobials in sub‐Saharan Africa.^19^, ^44^ These resistance patterns may be community‐acquired rather than hospital‐acquired as most study participants hardly seek medical attention. This is partly due to the long distance of hospitals from the endemic communities and poor education on antibiotic use. The study participants usually resort to the local licensed chemical store for medication compared to the community health centers to cut down the cost of treatment and alleviate them from painful filarial attacks.^45^ Elsewhere, the sensitivity trends among Gram‐negative bacteria corroborate with this present study's finding as most of the Gram‐negative bacteria were sensitive to ceftazidime, ciprofloxacin, gentamicin, cefuroxime, and clindamycin as well‐documented.^19^, ^38^, ^46^, ^47^, ^48^

The main limitation of this study is the possible underestimation of the total bacterial diversity among the study participants as the traditional culture method is biased toward only a few bacteria species that are “culturable” on artificial culture media. Additionally, the study was restricted to only bacteria species; thus, further studies could be conducted to understand the dynamics of other microbes such as fungi, viruses among people living with filarial lymphedema. Also, future prospects can possibly take a closer look at how “good” bacteria community could be essential in reversing the severity of filarial lymphedema. Despite these limitations, our data show a significant reduction of bacteria diversity toward the advanced stages of filarial lymphedema among individuals living with LF in the Ahanta West District in Ghana, indicating that, based on our data, a selective predominance of a few bacteria, which suppress the growth of other bacteria.

## 5 CONCLUSION

LF has been earmarked to be eradicated in 2020, however, this was not achieved. This could partly due to research scientist entirely focusing research on killing the adult worm or microfilariae with little attention to secondary bacterial infection which probably be aggravating the disease. This present study reveals that there is an existence of polymicrobial colonization in chronic wounds of individuals presenting with the infection and this has the potential in deteriorating the leg conditions of lymphedema. Moreover, the results of this study posit an emerging antimicrobial resistance trend among commonly used antibiotics in community endemic for LF in the Ahanta West District of Ghana.

## AUTHOR CONTRIBUTIONS

**Samuel O. Asiedu**: Formal analysis; methodology; writing – original draft; writing – review and editing. **Priscilla Kini**: Formal analysis; methodology; writing – review and editing. **Bill C. Aglomasa**: Formal analysis; methodology; writing – original draft. **Emmanuel K. A. Amewu**: Formal analysis; methodology; writing – review and editing. **Ebenezer Asiedu**: Formal analysis; methodology; writing – review and editing. **Solomon Wireko**: Formal analysis; methodology; writing – review and editing. **Kennedy G. Boahen**: Data curation; formal analysis; investigation; methodology; supervision. **Afiat Berbudi**: Writing – review and editing. **Augustina A. Sylverken**: Supervision; writing – review and editing. **Alexander Kwarteng**: Conceptualization; funding acquisition; investigation; methodology; project administration; resources; supervision; writing – review and editing.

## CONFLICTS OF INTEREST

All authors have read and approved the final version of the manuscript. Alexander Kwarteng (EDCTP‐TMA2016 1561) had full access to all the data in this study and takes complete responsibility for the integrity of the data and the accuracy of the data analysis.

## TRANSPARENCY STATEMENT

Alexander Kwarteng affirms that this manuscript is an honest, accurate, and transparent account of the study being reported; that no important aspects of the study have been omitted; and that any discrepancies from the study as planned (and, if relevant, registered) have been explained.

## Data Availability

The data sets used and/or analyzed during the current study are available from the corresponding author on reasonable request.
